# Comparative Financial Implications of Outpatient and Inpatient Service of the Psychiatric Department in General Hospital during the COVID-19 Pandemic in Taiwan: Case Report

**DOI:** 10.1192/j.eurpsy.2024.589

**Published:** 2024-08-27

**Authors:** W.-Y. Su, S.-C. Wang

**Affiliations:** ^1^National Yang Ming Chiao Tung University, Taipei City; ^2^Tao Yuan General Hospital, Ministry of Health and Welfare, Tao Yuan City, Taiwan, Province of China

## Abstract

**Introduction:**

The COVID-19 pandemic has stressed global healthcare systems, with Taiwan’s National Health Insurance (NHI) playing a crucial role in prevention and treatment. Like other countries, Taiwan grappled with managing the virus alongside regular healthcare services, resulting in notable financial strain on hospitals after COVID-19 pandemic.

**Objectives:**

This study explores the financial implications of the psychiatric department at a medical center in Taiwan, highlighting the changing dynamics of healthcare costs and revenue during this period.

**Methods:**

Data were collected monthly between January 2020 and September 2022, including the number of outpatient visits, inpatient patient-days, medical revenue, medical costs, and gross medical profit. Multivariate linear regression analysis confirmed the assumptions of the model and validated the findings.

**Results:**

Regression analysis revealed a significant correlation between the number of patients and financial indicators (USD^1^). Medical revenue **(Table. 1)**, grew by 82 USD for each outpatient visit(p<0.001, 95% CI:41–122), and grew by 70 USD for each inpatient-days(p=0.001, 95% CI:31–108). Medical costs **(Table. 2)**, increased by 59 USD for every inpatient-days (p=0.01, 95% CI:15–102). Finally, the gross medical profits **(Table. 3)** increased by 72 USD for each outpatient visit (p=0.003, 95% CI:27–117).

**Table 1.** Multiple linear regression analysis of the impact of medical service on medical revenue.

Revenue	Coef.	SE	t	*p*	[95% Conf. Interval]	
Outpatient (Visits)	82	20	4.136	.000	41	122
Inpatient (Patient Days)	70	19	3.664	.001	31	108

**Table 2.** Multiple linear regression analysis of the impact of medical service on medical cost.

Cost	Coef.	SE	t	*p*	[95% Conf. Interval]	
Outpatient (Visits)	9	22	0.422	.676	-36	55
Inpatient (Patient Days)	59	21	2.757	.010	15	102

**Table 3.** Multiple linear regression analysis of the impact of medical service on medical gross profit.

Gross Profit	Coef.	SE	t	*p*	[95% Conf. Interval]	
Outpatient (Visits)	72	22	3.261	.003	27	117
Inpatient (Patient Days)	11	21	0.501	.620	-33	54

^1^All values were converted from TWD to USD using the rate as of 2023/08/15.

In summary, outpatient visits significantly augmented revenue and gross profit, whereas inpatient days led to heightened revenue and costs.

**Conclusions:**

During the COVID-19 outbreak, healthcare systems, including those in Taiwan, were tested for unparalleled service challenges. This study found that while outpatient services boosted profits, rising inpatient admissions strained finances, given their higher costs and staffing needs. After the pandemic, psychiatric departments should reconsider resource allocation to balance expenses and revenues. Effective management is crucial for patient outcomes, emphasizing the need for quality care and fiscal control. Future research must focus on fortifying healthcare resilience.

**Disclosure of Interest:**

None Declared

